# *Dendrobium officinale* Kimura et Migo: A Review on Its Ethnopharmacology, Phytochemistry, Pharmacology, and Industrialization

**DOI:** 10.1155/2017/7436259

**Published:** 2017-03-12

**Authors:** Hanxiao Tang, Tianwen Zhao, Yunjie Sheng, Ting Zheng, Lingzhu Fu, Yongsheng Zhang

**Affiliations:** Zhejiang Chinese Medical University, 548 Binwen Rd., Binjiang District, Hangzhou, China

## Abstract

*Ethnopharmacological Relevance*. Dendrobii Officinalis Caulis, the stems of* Dendrobium officinale* Kimura et Migo, as a tonic herb in Chinese materia medica and health food in folk, has been utilized for the treatment of yin-deficiency diseases for decades.* Methods*. Information for analysis of* Dendrobium officinale* Kimura et Migo was obtained from libraries and Internet scientific databases such as PubMed, Web of Science, Google Scholar, ScienceDirect, Wiley InterScience, Ingenta, Embase, CNKI, and PubChem.* Results*. Over the past decades, about 190 compounds have been isolated from* Dendrobium officinale *Kimura et Migo. Its wide modern pharmacological actions in hepatoprotective effect, anticancer effect, hypoglycemic effect, antifatigue effect, gastric ulcer protective effect, and so on were reported. This may mainly attribute to the major and bioactive components: polysaccharides. However, other small molecule components require further study.* Conclusions*. Due to the lack of systematic data of* Dendrobium officinale*, it is important to explore its ingredient-function relationships with modern pharmacology. Recently, studies on the chemical constituents of* Dendrobium officinale* concentrated in crude polysaccharides and its structure-activity relationships remain scant. Further research is required to determine the* Dendrobium officinale* toxicological action and pharmacological mechanisms of other pure ingredients and crude extracts. In addition, investigation is needed for better quality control and novel drug or product development.

## 1. Introduction

*Dendrobium officinale* Kimura et Migo spreads in several countries over the world, such as Japan, United States, and Australia, and it distributes more widely in China [1] (Table 1). Modern pharmacology research has confirmed that* Dendrobium officinale* and its polysaccharide fraction possess anticancer, hepatoprotective, hypolipidemic, antifatigue, antioxidant, anticonstipation, hypoglycemic, gastric ulcer protective, and antihypertensive effects, immunoenhancement, and so on [2–4].

Since 1994, polysaccharides of* Dendrobium officinale* have been extracted and analyzed [5], and polysaccharides gradually became research focus of* Dendrobium officinale*. Recently, the chemical compositions and pharmacological effects of* Dendrobium officinale* have attracted more and more attention. In this review, from substantial data about* Dendrobium officinale*, approximately 190 monomer compositions and plenty of pharmacological researches studied in vivo and/or in vitro were summarized. In addition, the development of artificial cultivation of* Dendrobium officinale* accelerates the promotion of its industrialization in China. Patents and health care products were also improved by the combination of production and research mode.

## 2. Pharmaceutical Botany

**Figure 1:**
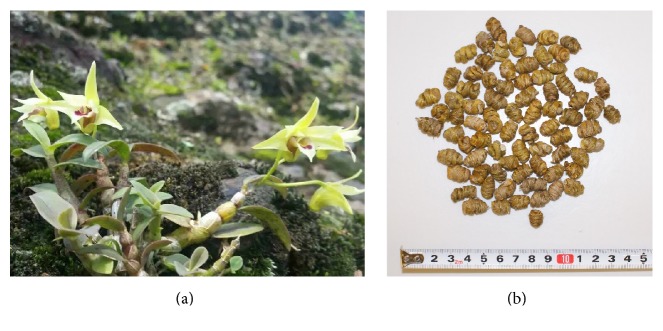
(a)* Dendrobium officinale* Kimura et Migo; (b)* tiepifengdou*.

Dendrobium, pertaining to the second largest family, Orchidaceae, includes approximately l,400 species worldwide distributed from tropical Asia to Oceania [6]. There are 74 species and 2 variants of genus* Dendrobium* Sw. in China [7]. Some of them can be processed as Dendrobii Caulis (Chinese: 石斛,* shihu*) of Chinese materia medica [8]. In 2005 Chinese Pharmacopoeia (ChP),* tiepishihu* (Chinese: *鐵皮*石斛) was one species of medicinal* shihu *called* Dendrobium candidum* Wall. ex Lindl. [9]; however this Latin name was later disputed and considered the synonym of* Dendrobium moniliforme* (L.) Sw. [10, 11]. In 2010 ChP,* tiepishihu* was renamed as* Dendrobium officinale* Kimura et Migo (aka* Dendrobium officinale*, Figure 1(a)). This Latin name was firstly denominated by two Japanese scholars in 1936 when they found the new species of* Dendrobium* in China [12]. Facilitating the preservation, the fibrous stems of* Dendrobium officinale* can be twisted into a spiral and dried as* tiepifengdou* (Figure 1(b)) or directly cut into sections and dried as well. What is more, in 2010 ChP,* tiepishihu* was initially isolated as one single medicine; its processed stem was called Dendrobii Officinalis Caulis (valid botanical name:* Dendrobium catenatum* Lindl., taxonomic classification and synonyms in Table 1); in the meanwhile,* Dendrobium nobile *Lindl.,* Dendrobium chrysotoxum* Lindl.*, Dendrobium fimbriatum *Hook., and their similar species were collectively referred to as* shihu* [13].

## 3. Ethnopharmacological Use

The stems of* Dendrobium officinale* are mainly used as health food in folk. There are a variety of methods to enjoy its fresh stems, for example, chewing, juicing, decocting, and making dishes [14]. In addition, combining with other tonic Chinese herbs is viable, such as “Panacis Quinquefolii Radix” (*xiyangshen*, American Ginseng), “Lycii Fructus” (*gouqizi*, Barbary Wolfberry Fruit), and “Dioscoreae Rhizoma” (*shanyao*, Rhizome of Common Yam) [15]. As reported, the combination of* Dendrobium officinale* and American Ginseng could strengthen cell-mediated immunity, humoral immunity, and monocyte-macrophages functions of mice [16].

The functions and indications of* tiepishihu* and* shihu* are quite similar in traditional Chinese medicine (TCM) [17, 18]. However, TCM physicians use* shihu* more frequently than* tiepishihu*. As precious medicinal materials, maybe the high price limits the application of* tiepishihu*. Because of the output promotion,* Dendrobium officinale* gradually got more applications, especially in recent decades. Research showed that low-grade fever after gastric cancer operations, atrophic gastritis, mouth ulcers, and diabetes were treated by the fresh* Dendrobium officinale* in TCM [19]. Combined with modern medicine,* Dendrobium officinale* can be used to treat many diseases including Sjögren's syndrome, gastric ulcer, alcoholic liver injury, chronic obstructive pulmonary disease, diabetes, obesity, rheumatoid arthritis, hypertensive stroke, cataract, weak constitution, or subhealth [2, 20–22]. However, these functions were from an earlier investigation and need further research to probe.

## 4. Phytochemistry

From* Dendrobium officinale*, at least 190 compounds by far were isolated, mainly including polysaccharides, phenanthrenes, bibenzyls, saccharides and glycosides, essential oils, alkaloids, and other compounds (Table 2).

### 4.1. Polysaccharides

Polysaccharides are the main component in dried* Dendrobium officinale*. Extraction and quantitative and qualitative analysis are described as follows, and the pharmacological effects of polysaccharides are in Table 3.

#### 4.1.1. Extraction

Generally, there are three methods that can isolate crude* Dendrobium officinale* polysaccharides (DOP): a solvent method (water extraction by alcohol sedimentation), a biological method (enzyme extraction), and a physical method (ultrasonic extraction and microwave extraction). Among the three, the solvent method is most popular. The physical method could be combined with the biological and solvent methods to accelerate the extraction process and promote the efficiency [23].

Since there are lipids, proteins, pigments, and other impurities after the process of separation of polysaccharides, other separation methods, such as ion-exchange chromatography, gel filtration chromatography, and HPLC, are needed to purify the crude polysaccharides [24]. However, polysaccharides are acknowledged as complex biological macromolecules; the ways and sequences of connections of the monosaccharides determine the difficulty of polysaccharides' analyses [25].

#### 4.1.2. Quantitative and Qualitative Analysis

The average content of neutral sugar in desiccated* Dendrobium officinale* is up to 58.3% [26]. Actually, different production origins, processing methods, growing years, and cultivated or wild and different parts of* Dendrobium officinale* are all related to polysaccharide content. Research showed that there were various polysaccharide contents of the same species cultivated in Guangdong with different origins from Yunnan, Zhejiang, and other provinces [27]. After drying each with different methods, DOP content was arranged as follows: hot air drying > vacuum drying > vacuum freeze drying > natural drying [28]. In addition, amounts of polysaccharide were greater in biennial* Dendrobium officinale* than annual or triennial ones [29], while the wild type contained more content compared to the cultivated type [30]. Research showed that polysaccharides distributing in different parts of* Dendrobium officinale* varied: middle stem > upper stem > lower stem > roots [31]. Therefore, accurate determination of polysaccharide content is of great significance.

**Figure 2:**
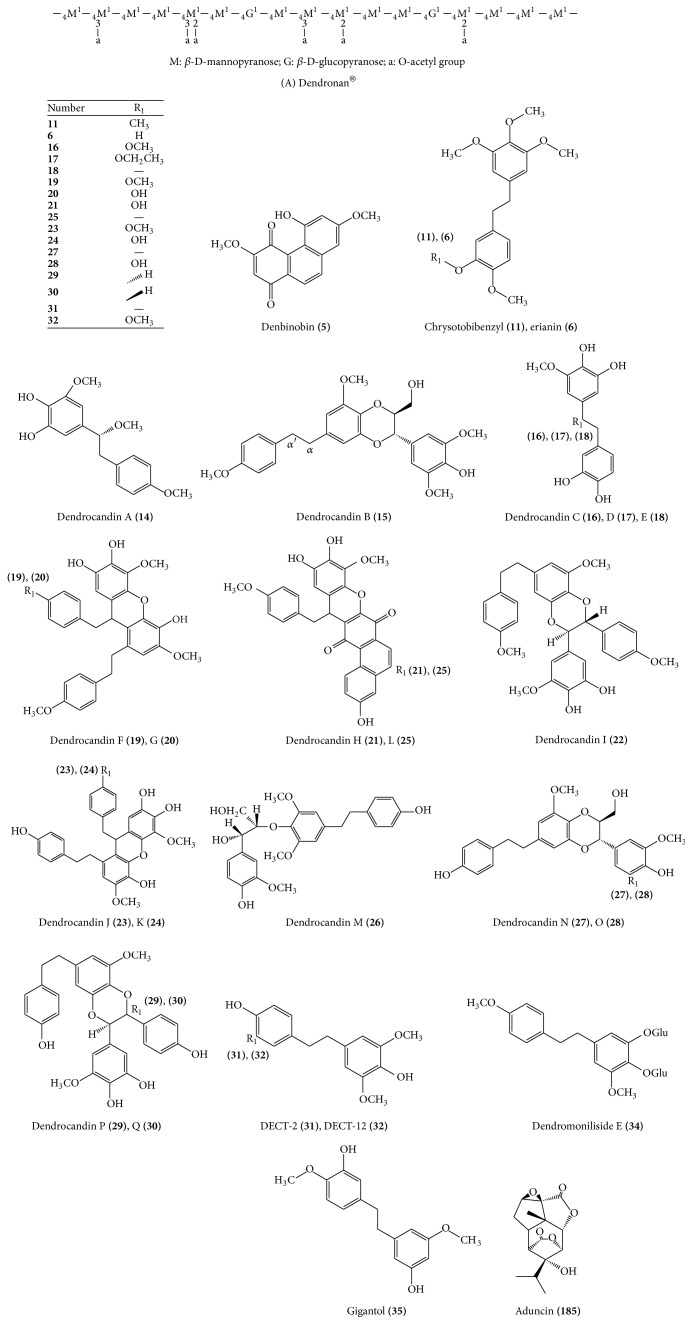
Some chemical structure of compounds isolated form* Dendrobium officinale* which have good potentials.

Fourier Transform Infrared Spectrometer (FT-IR), Gas Chromatography-Mass Spectrometer (GC-MS), and ^1^H and ^13^C NMR spectroscopies have been used to analyze the types of monosaccharide residues and the linkage sites of glycosidic bonds [32]. However, the research of polysaccharide's ratio of mannose to glucose, the existence of branches, and the substitution position of o-acetyl groups was inconsistent [33]. What is more, polysaccharides' pharmacological activities are strongly linked to the composition and content of their monosaccharides [26]. Therefore, further methods to explore the advanced structures and structure-activity relationships of* Dendrobium officinale* polysaccharides need to be established. Research showed that O-acetyl-glucomannan, Dendronan® (Figure 2, A), from* Dendrobium officinale* [34] has been isolated and was affirmed immunomodulatory activity in vitro [35] and in vivo [36], as well as improvement in colonic health of mice [37].

### 4.2. Phenanthrenes

From* Dendrobium officinale*, nine phenanthrenes** (1–9)** were isolated.* Dendrobium officinale* contains a kind of bibenzyl and a kind of phenanthrene identified in* D. chrysotoxum* before: chrysotobibenzyl** (11)** and erianin** (6)**. Both chrysotobibenzyl and erianin have antitumor effects. Erianin significantly inhibited the proliferation of HepG2 and Huh7 (human hepatoma cell lines) in vitro [38] and also prevented angiogenesis in human hepatoma Be17402 and human melanoma A375 in vivo [39]. IC_50_ of P388 murine leukemia cell treated by erianin in MTT assay was 0.11 *μ*M [40].

### 4.3. Bibenzyls

Bibenzyls** 10–35** were isolated from the stems of* Dendrobium officinale*. Among these compounds, 17 bibenzyls named Dendrocandins A–Q** (14–30)** (Figure 2) were extracted by Li et al. from 2008 to 2014. Next, DCET-2** (31)** could inhibit the proliferation of A2780 (human ovarian cancer cell line) cells, and DCET-12** (32)** inhibited BGC-823 (human gastric cancer cell lines) and A2780 cells [41]. DCET-18** (35)** also inhibited the migratory behavior and induced the apoptosis of non-small-cell lung cancer cells (human NCI-H460 cells) [42].

### 4.4. Saccharides and Glycosides

In the last few decades, at least 26 saccharides and glycosides have been found from* Dendrobium officinale*, (compounds** 63–88**). Among them, Dictamnoside A** (80)** showed immunomodulatory activity in mouse splenocyte assessed as stimulation of proliferation at 10 umole/L after 44 hours by MTT assay in the presence of Concanavalin A (ConA) [43].

### 4.5. Essential Oils

Compounds** 89–172**, isolated from* Dendrobium officinale*, comprise essential oils. The content of limonene** (123)** in the stem is 9.15% for the second; limonene is most abundant in the leaves, accounting for 38% [44]. Limonene contains anticancer properties with effects on multiple cellular targets in preclinical models [45].* Dendrobium officinale* containing a high content of (E)-2-hexenal** (106)** shows bactericidal activity against* Pseudococcus viburni*,* Pseudococcus affinis*,* Bemisia* sp., and* Frankliniella occidentalis* [46].

### 4.6. Alkaloids

Alkaloids are the earliest chemical compounds isolated from* Dendrobium* genus. Dendrobine, a kind of* Dendrobium* alkaloid accounting for 0.52%, was isolated initially in 1932 [47]. It was later proved to be the special content of* D. nobile* [48]. As reported, the alkaloid in* Dendrobium officinale* belongs to the terpenoid indole alkaloid (TIA) class, with its total content measured at approximately 0.02% [49]. However, the isolation and identification of one single kind of alkaloid have been rarely reported.

### 4.7. Others

Excepting ingredients above, phenols** (36–40)**, acids** (41–49)**, esters** (50–56)**, amides** (57–62)**, and other chemical constituents** (173–190)** were detected in* Dendrobium officinale* (Table 2), while their pharmacological effects remain to be excavated in the future.

## 5. Pharmacological Effects of* Dendrobium officinale*

Recently, more and more pharmacological actions of* Dendrobium officinale* were reported, such as hepatoprotective effect, anticancer effect, hypoglycemic effect, antifatigue effect, and gastric ulcer protective effect (Table 3).

### 5.1. Hepatoprotective Effect

The hepatoprotective capacity of* Dendrobium officinale* is always related to its antioxidant ability, especially in acute or chronic alcoholic liver injury. Research showed that* Dendrobium officinale* polysaccharides (DOP) accelerated the metabolism of serum TG and TC, while increased liver ADH and ALDH activities, which recovered disorders of lipid metabolism and accelerated excretion of alcohol and its metabolites [64]. In addition, ALT, AST, and TC of* Dendrobium officinale* treated mice models with chronic alcoholic liver injury were elevated compared to the normal groups [65]. Besides, compared to the model group of mice with acute alcoholic hepatic injury, fresh* Dendrobium officinale* and* tiepifengdou* groups could increase the SOD and reduce MDA of serum and liver tissue [66].

### 5.2. Anticancer Effect

The anticancer activity of* Dendrobium officinale* extract has been studied and proved, such as anti-HelaS3, anti-HepG2, and anti-HCT-116 in vitro, as well as anti-CNEl and CNE2 in vivo and in vitro [69]. Besides, DOP has manifested anticancer effects in mice with Lewis lung carcinoma (LLC). Its tumor inhibition rate was 8.5%–18.3% (*P* > 0.05); meanwhile, LLC mice spleen lymphocyte transformation and hemolysin levels (*P* < 0.05) were improved [67]. In cellular experiments, Liu's morphological analysis showed that DOP can inhibit the growth of human hepatoma cells (HepG2), human lung cancer cells (A549), human teratoma stem cells (NCCIT), and murine teratoma stem cells (F9) and promoted the proliferation of mouse spleen cells in vitro [68]. Research showed that two DOP fractions, DOP-1 and DOP-2, promoted splenocyte proliferation, enhanced NK cell-mediated cytotoxicity, and increased the phagocytosis and nitric oxide production of macrophages significantly (*P* < 0.05) [82]. Therefore, the anticancer effect of* Dendrobium officinale* may be accompanied by the activity of improving immune system.

### 5.3. Hypoglycemic Effect

Hypoglycemic activity, another important property of* Dendrobium officinale*, has been studied a lot. Oral administration of DOP decreased levels of fasting blood glucose (FBG) and glycosylated serum protein (GSP) and increased level of serum insulin in alloxan-induced diabetic mice. In addition, DOP attenuated the occurrence of oxidative stress in the liver and kidney of alloxan-induced diabetic mice by decreasing MDA levels, increasing GSH concentrations and antioxidative enzyme activities [70]. Therefore, DOP may regulate blood sugar levels through blood-lipid balance effects and antioxidative damage effects of liver and kidney.

*Dendrobium officinale* exhibited a significant hypoglycemic effect in adrenaline hyperglycemia mice and streptozotocin-diabetic (STZ-DM) rats [71]. In addition, TP (total polysaccharides, 100 mg/kg), TF (total flavonoids, 35 mg/kg), and TE (water extract, 6 g/kg) groups of* Dendrobium officinale* significantly downregulated the phosphorylation of JNK (Thr183/Tyr185) and upregulated the phosphorylation of AKT ser^473^ compared with the normal control group, which indicates that effective extracts of* Dendrobium officinale* have the effects of inhibiting JNK phosphorylation and promoting AKT ser^473^ phosphorylation [72].

### 5.4. Antifatigue Effect

The antifatigue effect of* Dendrobium officinale* was illustrated in vivo, when compared with the control groups;* Dendrobium officinale* could increase the glycogen stored in mice after exercise and decrease the level of serum urea and lactic acid accumulation (*P* < 0.05 or *P* < 0.01). In addition, it could upregulate the expression of ciliary neurotrophic factor (CNTF) mRNA (*P* < 0.05 or *P* < 0.01) [73]. In addition, compared to the control groups,* Dendrobium officinale* significantly increased carbon clearance indexes and burden swimming time and reduced the serum lactic acids [74]. In general, antifatigue effect of* Dendrobium officinal* was linked to the TCM use about enriching consumptive diseases, but its mechanism needs to be further clarified.

### 5.5. Gastric Ulcer Protective Effect

Research showed that* Dendrobium officinale* had a preventive effect of gastric injury caused by 60% ethanol-hydrochloric acid solution (ethanol was dissolved in 150 mM hydrochloric acid). Intragastric administration of SD rats with 200 mg/kg of* Dendrobium officinale* for two weeks decreased gastric secretion, IL-6 and TNF-*α* cytokine levels compared with the lower dose groups and the control groups. This concentration (200 mg/kg) had the strongest inhibitory effect of gastric injury (76.6% inhibition of gastric injury rate) [75]. After the successful establishment of the irritable gastric ulcer model (induced by cold water immersion) and chemical gastric ulcer model (indometacin-induced, 40 mg/kg, gavage) the ulcer index was calculated by Guth standard scoring method. Freshly squeezed* Dendrobium officinale* juice (containing crude drugs 0.5, 2 g/kg) showed significant declining irritable and chemical gastric ulcer model ulcer indexes (*P* < 0.01) [76]. However, gastric ulcer is a chronic disease, the security of long-term administration of* Dendrobium officinale* still uncertain.

### 5.6. Others

It is found that* Dendrobium officinale* had hypolipidemic and hypotensive effects. The fine powder solution of* Dendrobium officinale* (1.5, 3 g/kg) can reduce the levels of TG, TCHOL, and LDL-C in serum significantly and reduce the expression of TNF-*α* and IL-6 in ApoE^−/−^ mice. In addition, it could reduce areas of atheromatous plaque in aortic valve and arch in ApoE^−/−^ mice and then decrease the expression of TNF-*α* and IL-6 in aortic arch [77]. Research showed that stroke-prone spontaneously hypertensive (SHR-sp) rats' living days and survival rates were extended by DOP, and hypotensive effect of DOP was significantly better than the nonpolysaccharide ingredients of* Dendrobium officinale* [78].

Moreover,* Dendrobium officinale* was used to treat Sjögren's syndrome (SS) with dry eyes and mouth due to impaired lacrimal and salivary glands [80]. Research showed that DOP could suppress the progressive lymphocytes infiltration and apoptosis and balance the disorders of proinflammatory cytokines in the submandibular gland (SG) in vivo. Further, DOP ameliorated the abnormalities of aquaporin-5 (AQP-5) that was supported by in vitro study on A-253 cell line and maintained its function of saliva secretion [79].

Meanwhile, research showed that the DOP group demonstrated higher average scores and an improved average quality of hair growth of C57BL/6J mice (5.0 g/L, the solution was ultra-pure water, each 0.2 mL, smeared for 21 d), compared with the control group. Besides, DOP significantly increased HaCaT cells survival rate and the VEGF mRNA expression levels compared with the control group [80].

Besides, the* Dendrobium officinale* had an obvious effect on the molecular diversity of intestinal* Lactobacillus* in mice model (irregular diet for 8 d after gavage of folium senna water decoction for 7 d) with constipation resulting from spleen deficiency (TCM syndrome type) [81].* Dendrobium officinale* also can regulate the digestive function in carbon-induced constipated mice models [83].

## 6. The Toxicology of* Dendrobium officinale*

After acute toxicity test, genetic toxicity test (Ames test, micronucleus test of bone marrow, and sperm shape abnormality test in mice) and 90-day feeding test in rats, the results showed that protocorms of* Dendrobium officinale* were without toxicity, genetic toxicity and mutagenicity within the scope of the test dose [84]. Furthermore, the 15% decoction of* Dendrobium officinale* was used for mice sperm malformation test, Ames test, and micronucleus test of bone marrow in mice, and all test results were negative. In addition, the acute oral toxicity test showed that the highest dose was 10 g/kg b.w. which belonged to the actual nontoxic category [85]. However, in the pesticide safety aspect, the* Dendrobium officinale* still need more stringent quality control [86].

## 7. The Industrialization of* Dendrobium officinale*

Due to the special trophic mode, seed germination of* Dendrobium officinale* needs the root symbiotic bacteria [87, 88]; the reproduction of wild* Dendrobium officinale* is limited with low natural reproductive rates. The past three decades witnessed excess herb-gathering of* Dendrobium officinale* destroying much of the wild habitat resource in China [89]. In 1987,* Dendrobium officinale* was on the list of national third-level rare and endangered plants [90]. However, artificial cultivation based on tissue culture technology significantly prompted the yield of* Dendrobium officinale*.

Recently, increased awareness of the tonic therapeutic effect of* Dendrobium officinale* has increased the demand and as a consequence the price [91]. However, the high-profit margin of* Dendrobium officinale* has already led to an increase in the market for counterfeits and adulterants [92] mainly by other confusable species of* Dendrobium *[91]. The appearance of* Dendrobium officinale* and other species of* Dendrobium* is very similar, especially after processing into* “tiepifengdou”*; it is difficult to distinguish them through morphological identification [93]. It is apparent that* Dendrobium officinale* germplasm resources' separation and purification determine its characters and quality [94]. In terms of microscopic identification,* Dendrobium officinale* could be identified by vascular bundle sheath observed under the fluorescence microscopy and the distribution of raphides under normal light microscopy [95]. In addition, the taxonomy, phylogeny, and breeding of* Dendrobium* species have made great progress as the advance of molecular markers in the past two decades [96].

Under such circumstances, pharmacognosy [97], DNA molecule marker technologies including Amplified Fragment Length Polymorphism (AFLP) [98], Inter Simple Sequence Repeat (ISSR) [99], Start codon targeted (SCoT) and target region amplification polymorphism (TRAP) [100], and sequence related amplified polymorphism (SRAP) analysis [101], and so on have been applied to identify* Dendrobium officinale* from other Dendrobium species. Further, Yunnan province has completed* Dendrobium officinale* fine gene map and found more than 48,200 protein-coding genes [102]. Therefore, the identification of* Dendrobium officinale* is more precise due to the gene technology, but genetic fingerprints are difficult to evaluate the quality, so the gene technology combining with the physical and chemical identification is necessary.

In recent years, the development of* Dendrobium officinale* health care products is promising [15]. Many related patented products are being registered or have been authorized, including* Dendrobium officinale* ingredients such as antiaging compounds [103], lactobacillus drink [104], antiasthenopia eye ointment [105], immunoenhancement compounds [106], hypotensive extractive [107], and hypoglycemic and hypolipidemic compounds [108]. Some of these patents have been put into production like* “tiepifengdou*,*”* one of the most famous processed products of* Dendrobium officinale* [101]. However, there are few medicines of* Dendrobium officinale* [109] besides* tiepishihu *of Chinese materia medica in TCM.

**Figure 3:**
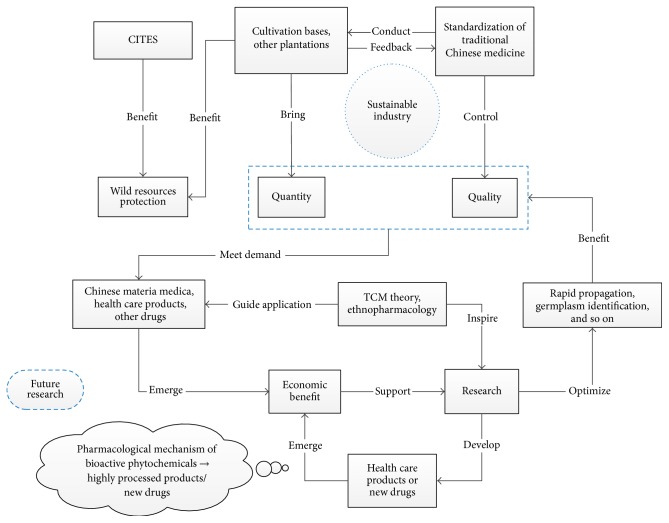
The industry and research* status quo* and future perspectives.

In summary, the TCM theory and ethnopharmacology could inspire modern researches committed to yield and quality control of* Dendrobium officinale*, while some standards or laws (such as ISO/TC 249 and CITES) regulated artificial cultivation and protected wildlife resources. Finally, industrialization will drive further researches and is conducive to develop deep-processing products, especially the insufficient new drugs, which will also promote the industrialization and form a virtuous circle simultaneously (Figure 3).

## 8. Conclusion

*Dendrobium officinale*, one of the most famous species of* Dendrobium*, has long been regarded as precious herbs and health foods applied in TCM and in folk. In this paper, the ethnopharmacology, phytochemistry, pharmacology, and industrialization of* Dendrobium officinale* were summarized. In recent years, the interest of exploring ethnopharmacology-based bioactive constituents of* Dendrobium officinale* has increased considerably. Rapid propagation technology has gradually matured, so yield is no longer the bottleneck of* Dendrobium officinale *development. In addition, the confusable identification and uneven quality still exist, and better quality control standards under modern researches are necessary.

Consequently, the following points deserve further investigation. (1) Polysaccharides are the main composition in* Dendrobium officinale* with numerous pharmacological researches, but investigation related to its structure-activity relationships remains scant. (2) The pharmacological effects of* Dendrobium officinale* crude extract and polysaccharide were similar, indicating whether other active ingredients were lost during extraction. (3) There is no enough systemic data about toxicology of* Dendrobium officinale*. (4) How does TCM theory play a role in* Dendrobium officinale* further in-depth development as theoretical guidance and inspiration source.

Over all, further studies at the molecular level are needed to promote the exploration of chemical compositions and pharmacological mechanisms. In addition, the industrialization of* Dendrobium officinale* not only protected the germplasm resources but also attracted a large quantity of researches in China, which makes the innovation of* Dendrobium officinale* novel drug and product a promising prospect.

## Figures and Tables

**Table 1:** The taxonomic classification, names, and distribution of *Dendrobium officinale* Kimura et Migo.

Synonyms	*Dendrobium catenatum* Lindl., Gen. Sp. Orchid. Pl.: 84 (1830).	*Dendrobium stricklandianum *Rchb.f., Gard. Chron., n.s., 7: 749 (1877).	*Callista stricklandiana* (Rchb.f.) Kuntze, Revis. Gen. Pl. 2: 655 (1891).	*Dendrobium tosaense* Makino, J. Bot. 29: 383 (1891).	*Dendrobium pere-fauriei *Hayata, Icon. Pl. Formosan. 6: 70 (1916).	*Dendrobium tosaense var. pere-fauriei* (Hayata) Masam., J. Soc. Trop. Agric. 4: 196 (1933).	*Dendrobium officinale* Kimura & Migo, J. Shanghai Sci. Inst. 3: 122 (1936).
Taxonomic classification	Species 2000 & ITIS Catalogue of Life: Plantae > Tracheophyta > Liliopsida > Asparagales > Orchidaceae > Dendrobium > *Dendrobium catenatum* Lindl.	IUCN Red List: Plantae > Tracheophyta > Liliopsida > Orchidales > Orchidaceae > Dendrobium > *Dendrobium officinale*	NCBI Taxonomy: Eukaryota > Viridiplantae > Streptophyta > Streptophytina > Embryophyta > Tracheophyta > Euphyllophyta > Spermatophyta > Magnoliophyta > Mesangiospermae > Liliopsida > Petrosaviidae > Asparagales > Orchidaceae > Epidendroideae > Epidendroideae incertae sedis > Dendrobiinae > Dendrobium > *Dendrobium catenatum*	Taxonomic Hierarchy of COL-China 2012: Plantae > Angiospermae > Monocotyledoneae > Microspermae > Orchidaceae > Dendrobium > *Dendrobium tosaense*	Tropicos resource: Equisetopsida C. Agardh > Asparagales Link > Orchidaceae Juss. > Callista Lour. > *Callista stricklandiana *Kuntze	Tropicos resource: Equisetopsida C. Agardh > Asparagales Link > Orchidaceae Juss. > Dendrobium Sw. > *Dendrobium officinale *Kimura & Migo	
Distribution	Japan > Kyushu	United States > Missouri > Saint Louis City		Australia > New South Wales		China > Taiwan, Anhui, Zhejiang, Fujian, Guangxi, Sichuan, Yunnan, Xizang, etc.	
Common name	*鐵皮*石斛 *(tiepishihu)*			黃石斛 *(huangshihu)*			
Reference	http://www.kew.org/ http://www.theplantlist.org/		http://www.catalogueoflife.org/ http://www.tropicos.org/	http://www.gbif.org/ http://www.eol.org/	http://frps.eflora.cn/	[50] [51]	[10]

**Table 2:** Chemical components in *Dendrobium officinale*.

Number	Compounds	PubChem CID	Ref.
Phenanthrenes			
1	2,3,4,7-Tetramethoxyphenanthrene		[52]
2	2,5-Dihydroxy-3,4-dimethoxyphenanthrene		[52]
3	1,5-Dicarboxy-1,2,3,4-tetramethoxyphenanthrene		[52]
4	2,4,7-Trihydroxy-9,10-dihydrophenanthrene	21678577	[41]
5	Denbinobin	10423984	[41]
6	Erianin	356759	[53]
7	1,5,7-Trimethoxyphenanthrene-2,6-diol	11779542	[52]
8	3,4-Dimethoxyphenanthrene-2,7-diol	158975	[52]
9	2,4-Dimethoxyphenanthrene-3,5-diol	44445443	[52]
Bibenzyls			
10	3,4-Dihydroxy-5,4′-dimethoxybibenzyl		[54]
11	Chrysotobibenzyl	3086528	[53]
12	4′,5-Hydroxy-3,3′-dimethoxybenzyl		[55]
13	3,4′-Dihydroxy-5-methoxybibenzyl		[41]
14	Dendrocandin A	102476850	[56]
15	Dendrocandin B	91017475	[56]
16	Dendrocandin C	25208514	[56]
17	Dendrocandin D	25208516	[56]
18	Dendrocandin E	25208515	[56]
19	Dendrocandin F		[56]
20	Dendrocandin G		[56]
21	Dendrocandin H		[56]
22	Dendrocandin I	101481782	[56]
23	Dendrocandin J		[56]
24	Dendrocandin K		[56]
25	Dendrocandin L		[56]
26	Dendrocandin M		[56]
27	Dendrocandin N		[56]
28	Dendrocandin O		[56]
29	Dendrocandin P		[56]
30	Dendrocandin Q		[56]
31	4,4′-Dihydroxy-3,5-dimethoxybibenzyl	442701	[56]
32	4,4′-Dihydroxy-3,3′,5-trimethoxybibenzyl	176096	[56]
33	3,4,4′-Trihydroxy-5-methoxybibenzyl		[57]
34	Dendromoniliside E		[41]
35	Gigantol	3085362	[54]
Phenols			
36	4-(3,5-Dimethoxyphenethyl) phenol		[57]
37	3-(4-Hydroxyphenethyl)-5-methoxyphenol		[57]
38	5-(3-Hydroxyphenethyl)-2-methoxyphenol		[57]
39	4-(4-Hydroxyphenethyl)-2,6-dimethoxyphenol		[57]
40	4-(4-Hydroxy-3-methoxyphenethyl)-2,6-dimethoxyphenol		[57]
Acids			
41	*p-*Hydroxy-coumaric acid	637542	[55]
42	*p-*Hydroxybenzene propanoic acid		[55]
43	Hexadecanoic acid	985	[41]
44	Heptadecanoic acid	10465	[41]
45	Syringic acid	10742	[58]
46	Vanillic acid	8468	[58]
47	*p-*Hydroxy-phenylpropionic acid	10394	[58]
48	Ferulic acid	445858	[58]
49	4-Hydroxybenzoic acid	135	[58]
Esters			
50	*p-*Hydroxyl-trans-cinnamic acid myricyl ester		[41]
51	Trans-ferulic acid octacosyl ester		[41]
52	*p-*Hydroxyl-*cis*-cinnamic acid myricyl ester		[41]
53	Dihydroconiferyl dihydro-*p-*cumarate		[58]
54	Cis-3-(4-Hydroxy-3-methoxy-phenyl)-acrylic acid octacosyl ester		[57]
55	Trans-3-(4-Hydroxy-3-methoxy-phenyl)-acrylic acid octacosyl ester		[57]
56	4-[2-(4-Methoxy-phenyl)-ethyl-6]-oxo-6H-pyran-2-carboxylic acid methyl ester		[57]
Amides			
57	N-Trans-feruloyltyramine	5280537	[55]
58	*cis*-Feruloyl *p-*hydroxyphenethylamine		[55]
59	Trans cinnamyl p-hydroxyphenethylamine		[55]
60	N-*p-*Coumaroyltyramine	5372945	[58]
61	Dihydroferuloyltyramine	90823368	[58]
62	4-Hydroxy-N-[2-(4-hydroxyphenyl)ethyl]benzenepropanamide		[58]
Saccharides and glycosides			
63	4-Allyl-2,6-dimethoxy phenyl glycosidase		[55]
64	Adenosine	60961	[41]
65	Uridine	6029	[41]
66	Vernine	46780355	[41]
67	Apigenin-7-O-*β*-D-glucoside	5280704	[59]
68	Icariol-A2-4-O-*β*-D-glucopyranoside	6439218	[59]
69	(+)-Syringaresinol-O-*β*-D-pyranglucose		[59]
70	Dihydrosyringin	71720642	[58]
71	Vicenin 3	44257698	[55]
72	Isoschaftoside	3084995	[55]
73	Schaftoside	442658	[55]
74	Vicenin 2	442664	[55]
75	Apigenin 6-C-*α*-L-arabinopyranosyl-8-C-*β*-D-xylopyranoside		[55]
76	Apigenin 6-C-*β*-D-xylopyranosyl-8-C-*α*-L-arabinopyranoside		[55]
77	Vincenin 1	44257662	[55]
78	2-Methoxyphenol-O-*β*-D-apiofuromosyl-(1→2)-*β*-D-glucopyranoside		[60]
79	3,4,5-Trimethoxyphenyl-1-O-*β*-D-apiose-(1→2)-*β*-D-glucoside		[60]
80	Dictamnoside A	44560015	[61]
81	Leonuriside A	14237626	[60]
82	(1′R)-1′-(4-Hydroxy-3,5-dimethoxylphenyl) propan-1′-ol 4-O-*β*-D-glucopyranoside		[60]
83	Syringaresinol-4,4′-O-bis-*β*-D-glucoside		[60]
84	(+)-Syringaresinol-4-*β*-D-monoglucoside		[60]
85	(+)-Lyoniresinol-3a-O-*β*-glucopyranoside		[60]
86	3,5-Dimethoxy-4-hydroxylphenyl-1-O-*β*-D-pyranglucose		[59]
87	7-Methoxycoumarin-6-O-*β*-D-pyranglucose		[59]
88	Sucrose	5988	[41]
Essential oils			
89	Methyl acetate	6584	[44]
90	Carbon disulfide	6348	[44]
91	Hexane	8058	[44]
92	Ethyl acetate	8857	[44]
93	2-Methyl-propanol	6560	[44]
94	3-Methyl-butanal	11552	[44]
95	2-Methyl-butanal	7284	[44]
96	2-Pentanone	7895	[44]
97	Pentanal	8063	[44]
98	3-Pentanone	7288	[44]
99	3-Methyl-1-butanol	31260	[44]
100	2-Methyl-1-butanol	8723	[44]
101	2-Methyl-3-pentanone	11265	[44]
102	1-Pentanol	6276	[44]
103	(Z)-3-Hexanone		[44]
104	2-Hexanone	11583	[44]
105	Hexanal	6184	[44]
106	(E)-2-Hexenal	5281168	[44]
107	(E)-3-Hexanol		[44]
108	(Z)-3-Hexenol		[44]
109	1-Hexanol	8103	[44]
110	Styrene	7501	[44]
111	(E)-2-Heptenal	5283316	[44]
112	Benzaldehyde	240	[44]
113	1-Heptanol	8129	[44]
114	1-Octen-3-one	61346	[44]
115	1-Octen-3-ol	18827	[44]
116	6-Methyl-5-hepten-2-one	9862	[44]
117	2-Pentyl-furan	19602	[44]
118	Decane	15600	[44]
119	Octanal	454	[44]
120	(Z)-3-Hexenyl acetate	5363388	[44]
121	Hexyl acetate	8908	[44]
122	*p-*Cymene	7463	[44]
123	Limonene	22311	[44]
124	3-Octen-2-one	15475	[44]
125	(E)-*β*-Ocimene	5281553	[44]
126	(E)-2-Octenal	5283324	[44]
127	Ethyl benzyl ether	10873	[44]
128	1-Phenyl-ethanone	7410	[44]
129	(E)-2-Nonen-1-ol	5364941	[44]
130	1-Octanol	957	[44]
131	Nonanal	31289	[44]
132	2-Heptenyl acetate	85649	[44]
133	(E)-2-Nonenal	5283335	[44]
134	Nonenol	85445514	[44]
135	2-Terpineol		[44]
136	Dodecane	8182	[44]
137	2-Octenyl acetate		[44]
138	Decyl aldehyde	8175	[44]
139	Benzylacetone	17355	[44]
140	(E)-2-Decenal	5283345	[44]
141	1-Dodecanol	8193	[44]
142	Tridecane	12388	[44]
143	*α*-Cubebene	86609	[44]
144	*β*-Bourbonene	62566	[44]
145	Tetradecane	12389	[44]
146	*α*-Cedrene	442348	[44]
147	Zingiberene	92776	[44]
148	Geranyl acetone	19633	[44]
149	2-Methyl tridecane		[44]
150	AR-Curcumene	92139	[44]
151	Pentadecane	12391	[44]
152	*β*-Bisabolene	403919	[44]
153	(+)-D-Cadinene	441005	[44]
154	5-Phenyl-decane		[44]
155	4-Phenyl-decane		[44]
156	Hexadecane	11006	[44]
157	*α*-Cedrol	65575	[44]
158	5-phenyl-undecane		[44]
159	4-phenyl-undecane		[44]
160	Heptadecane	12398	[44]
161	Pristane	15979	[44]
162	6-Phenyl-dodecane	17629	[44]
163	5-Phenyl-dodecane	17630	[44]
164	4-Phenyl-dodecane	17631	[44]
165	6,10,14-Trimethyl-2-pentadecanone	10408	[44]
166	Butyl phthalate	3026	[44]
167	Isobutyl phthalate	6782	[44]
168	Sandaracopimaradiene	440909	[44]
169	Hentriacontanol	68345	[62]
170	Citronellol	8842	[55]
171	Citrusin C	3084296	[59]
172	Coniferyl alcohol	1549095	[55]
Others			
173	2,6-Dimethoxycyclohexa-2,5-diene-1,4-dione		[63]
174	(24R)-6-*β*-Hydroxy-24-ethlcholest-4-en-3-one		[63]
175	Dendroflorin	44418788	[63]
176	Friedelin	91472	[63]
177	3-Ethoxy-5-hydroxy-7-methoxy-1,4-phenanthra-quinone		[63]
178	*β*-sitosterol	222284	[62]
179	5-Hydroxymethyl furfural	237332	[41]
180	Syringaldehyde	8655	[58]
181	3-O-Methylgigantol	10108163	[54]
182	Syringaresinol	332426	[55]
183	5*α*,8*α*-Epidioxy-24(R)-methylcholesta-6,22-dien-3*β*-ol		[63]
184	(−)-Secoisolariciresinol	65373	[60]
185	Aduncin	101316879	[41]
186	*β*-Daucosterol	5742590	[62]
187	(−)-Loliolide	12311356	[41]
188	Naringenin	932	[61]
189	3′,5,5′,7-Tetrahydroxyflavanone		[61]
190	Dihydrogen resveratrol		[41]

**Table 3:** The pharmacological effects of *Dendrobium officinale.*

Effect	Tested material	In vivo/in vitro	Adm.	Conc.	Observations	Ref.
Hepatoprotective effect	Arabinose : mannose : glucose : galactose = 1.26 : 4.05 : 32.05 : 3.67	In vivo	Gavage	50, 100, and 200 mg/kg	Increased the weight in mice	[64]
		In vivo	Gavage	200 mg/kg	Increased liver coefficient in mice	
		In vivo	Gavage	50, 100, and 200 mg/kg	Decreased mice serum ALT, AST, ALP activity, and TBIL levels; increased serum HDL-C; decreased LDL-C levels; accelerated metabolism of serum TG and TC; increased liver ADH, ALDH activities; inhibited mRNA expression of P4502E1, TNF-*α*, and IL-1*β*	
	Arabinose : mannose : glucose : galactose = 1.26 : 4.05 : 32.05 : 3.67	In vivo	Gavage	100 and 200 mg/kg	Increased GR activity and GSH-Px activity in mice	[64]
	Original extract solution	In vivo	Gavage	3 g/kg	Reduced chronic alcoholic liver injured mice's serum ALT, AST, and TC levels	[65]
	Original extract solution	In vivo	Gavage	6 g/kg	Reduced serum TC level in mice	
	*Tiepifengdou *original extract solution	In vivo	Gavage	0.45, 0.9, and 1.35 g/kg	Reduced serum AST levels in mice	
	Original extract solution	In vivo	Gavage	3 g/kg	Increased acute alcoholic hepatic injured mice's SOD of serum and liver and DSG-Px of liver	[66]
	*Tiepifengdou *original extract solution	In vivo	Gavage	0.45, 0.90, and 1.35 g/kg	Increased acute alcoholic hepatic injured mice's SOD of serum and liver and DSG-Px of liver	
	Original extract solution	In vivo	Gavage	3 and 6 g/kg	Reduced MDA of serum and liver in mice	
	*Tiepifengdou *original extract solution	In vivo	Gavage	0.45 and 0.90 g/kg	Reduced MDA of serum and liver in mice	
	Original extract solution	In vivo	Gavage	9 g/kg	Reduced serum MDA in mice	

Anticancer effect	Water extraction by alcohol sedimentation, extraction rate (in dry herb): 19.2%	In vivo	Gavage	10 and 20 g/kg	Increased the level of carbon clearance indexes and NK cells activity of LLC mice (*P* < 0.05)	[67]
		In vivo	Gavage	20 g/kg	Improved the LLC mice's spleen lymphocyte transformation and hemolysin levels (*P* < 0.05)	
	Water extraction by alcohol sedimentation	In vitro	—	100, 200, and 400 *μ*g/mL	Inhibited the growth of human hepatoma cells (HepG2), human lung cancer cells (A549), and human teratoma stem cells (NCCIT)	[68]
		In vitro	—	200 and 400 *μ*g/mL	Inhibited murine teratoma stem cells (F9) and promoted their apoptosis	
		In vitro	—	100 *μ*g/mL	Promoted the proliferation of mouse spleen cells	
	Water extraction and alcohol precipitation	In vivo	Gavage	—	Inhibited the growth transplantation tumor (CNE1 and CNE2) of NPC nude mice	[69]
		In vitro	—	128 and 256 mg/L	Inhibited the proliferation and induced the apoptosis of CNE1 and CNE2 cells; activated caspase-3; declined Bcl-xL, Mcl-1 protein levels	

Hypoglycemic effect	DOP: 2-O-acetylglucomannan consisted of Man, Glc, and Ara in the molar ratio of 40.2 : 8.4 : 1.0	In vivo	Gavage	200, 100, and 50 mg/kg	Decreased levels of fasting blood glucose (FBG) and glycosylated serum protein (GSP); increased level of serum insulin in alloxan induced diabetic mice; attenuated the occurrence of oxidative stress in the liver and kidney of alloxan-induced diabetic mice (decreased MDA levels; increased GSH concentrations and antioxidative enzyme activities)	[70]
	Extract, crude drug 1.8 g/g	In vivo	Gavage	0.125 and 0.25 g/kg	Reduced STZ-DM rats' blood glucose and glucagon levels; enhanced the number of islet *β* cells; declined the number of islet *α* cells	[71]
		In vivo	Gavage	0.5 and 1.0 g/kg	Decreased blood glucose and increased liver glycogen content in adrenaline induced hyperglycemia rats	
	Total polysaccharide: 43.1%, total flavonoids: 19.6%, crude drug 1 g/mL	In vivo	Gavage	TP (total polysaccharides, 100 mg/kg), TF (total flavonoids, 35 mg/kg), and TE (water extract, 6 g/kg)	Significantly downregulated the phosphorylation of JNK (Thr^183^/Tyr^185^) and upregulated the phosphorylation of AKT ser^473^ in rat	[72]

Antifatigue effect	Hot water reflux and cellulase lixiviating extraction	In vivo	Gavage	1.5 and 4.5 g/kg	Increased mice's glycogen store after exercise fatigue	[73]
	Hot water reflux and cellulase lixiviating extraction	In vivo	Gavage	0.75, 1.5, and 4.5 g/kg	Decreased the level of serum urea and lactic acid accumulation; upregulated the expression of CNTF mRNA in mice	
	Hot water extraction then filtration	In vivo	Gavage	0.75 mg/kg	Increased carbon clearance indexes from 0.025 to 0.034 in mice	[74]
		In vivo	Gavage	3 and 6 mg/kg	Extended burden swimming time of mice; reduced serum lactic acids of mice	[74]

Gastric ulcer protective effect	Lyophilization then hot-water extraction, evaporation	In vivo	Gavage	200 mg/kg	Decreased SD rats' gastric secretion, IL-6, and TNF-*α* cytokine levels; had 76.6% inhibition of gastric injury rate	[75]
	Squeezing then filtration	In vivo	Gavage	0.5 and 2 g/kg	Declined irritable and chemical gastric ulcer model's ulcer indexes in mice	[76]

Others	Dry powder	In vivo	Gavage	1.5 and 3 g/kg	Reduced ApoE^−/−^ mice's TG, TCHOL, LDL-C levels, and expression of TNF-*α* and IL-6 in serum; reduced areas of atheromatous plaque in aortic valve and arch in ApoE^−/−^ mice; then decreased expression of TNF-*α* and IL-6 in aortic arch	[77]
	Aqueous extract	In vivo	Gavage	0.25, 0.5, and 1.0 crude drug g/kg	Extended Stroke-prone spontaneously hypertensive (SHR-sp) rats' blood pressure, living days, and survival rate	[78]
	Water extraction by alcohol sedimentation; mannose : glucose : galactose : arabinose : xylose : glucuronic acid = 10 : 0.25 : 1.2 : 4.7 : 1.3 : 1.4	In vivo	—	20 mg/mL	Inhibited Bax/Bal-2 ratio and caspase-3 expression; decreased expression of cytokines (TNF-*α*, IL-1*β*, and IL-6) and activity of MMP-9 in mice	[79]
	Water extraction by alcohol sedimentation; mannose : glucose : galactose : arabinose : xylose : glucuronic acid = 10 : 0.25 : 1.2 : 4.7 : 1.3 : 1.4	In vitro	—	0.1, 1.0, and 10 *μ*g/mL	Ameliorated the abnormalities of aquaporin-5 (AQP-5) on A-253 cell	[79]
	Water extract and alcohol precipitate, extraction rate: 29.87%	In vivo	Smear	5.0 g/L	Increased average score and average quality of hair growth of C57BL/6J mice	[80]
		in vitro	—	0.1, 1.0, and 5.0 mg/L	Increased HaCaT cells survival rate and the VEGF mRNA expression level	
	Dry power decoction then concentration: traditional decoction or dry power steep in hot water: ultra-fine powder decoction	In vivo	Gavage	—	Increased Shannon index and Brillouin index in mice with constipation and improved their molecular diversity of intestinal *Lactobacillus*	[81]

## Abbreviations

ADH: Antidiuretic hormone
ALDH: Acetaldehyde dehydrogenase
b.w.: Body weight
ChP: Chinese Pharmacopoeia
CITES: Convention of International Trade in Endangered Species of Wild Fauna and Flora
ConA: Concanavalin A
*D*.: *Dendrobium*
DOP: *Dendrobium officinale* polysaccharides
GC: Gas Chromatography
GSH-PX: Glutathione peroxidase
HDL: High-density lipoprotein
HPLC: High Performance Liquid Chromatography
LLC: Lewis lung carcinoma
MDA: Malondialdehyde
MMP: Matrix metalloproteinase
MS: Mass Spectrometer
MTT: 3-(4,5-Dimethyl-2-thiazolyl)-2,5-diphenyl-2-H-tetrazolium bromide
NMR: Nuclear Magnetic Resonance
SOD: Superoxide dismutase
SS: Sjögren's syndrome
TC: Total cholesterol
TCM: Traditional Chinese medicine
TG: Triglyceride
VEGF: Vascular endothelial growth factor.
